# Implications of Diet for the Extinction of Saber-Toothed Cats and American Lions

**DOI:** 10.1371/journal.pone.0052453

**Published:** 2012-12-26

**Authors:** Larisa R. G. DeSantis, Blaine W. Schubert, Jessica R. Scott, Peter S. Ungar

**Affiliations:** 1 Department of Earth and Environmental Science, Vanderbilt University, Nashville, Tennessee, United States of America; 2 Don Sundquist Center of Excellence in Paleontology and Department of Geosciences, East Tennessee State University, Johnson City, Tennessee, United States of America; 3 Department of Sociology and Anthropology, University of Arkansas at Little Rock, Little Rock, Arkansas, United States of America; 4 Department of Anthropology, University of Arkansas, Fayetteville, Arkansas, United States of America; University College London, United Kingdom

## Abstract

The saber-toothed cat, *Smilodon fatalis*, and American lion, *Panthera atrox*, were among the largest terrestrial carnivores that lived during the Pleistocene, going extinct along with other megafauna ∼12,000 years ago. Previous work suggests that times were difficult at La Brea (California) during the late Pleistocene, as nearly all carnivores have greater incidences of tooth breakage (used to infer greater carcass utilization) compared to today. As Dental Microwear Texture Analysis (DMTA) can differentiate between levels of bone consumption in extant carnivores, we use DMTA to clarify the dietary niches of extinct carnivorans from La Brea. Specifically, we test the hypothesis that times were tough at La Brea with carnivorous taxa utilizing more of the carcasses. Our results show no evidence of bone crushing by *P. atrox*, with DMTA attributes most similar to the extant cheetah, *Acinonyx jubatus*, which actively avoids bone. In contrast, *S. fatalis* has DMTA attributes most similar to the African lion *Panthera leo*, implying that *S. fatalis* did not avoid bone to the extent previously suggested by SEM microwear data. DMTA characters most indicative of bone consumption (i.e., complexity and textural fill volume) suggest that carcass utilization by the extinct carnivorans was not necessarily more complete during the Pleistocene at La Brea; thus, times may not have been “tougher” than the present. Additionally, minor to no significant differences in DMTA attributes from older (∼30–35 Ka) to younger (∼11.5 Ka) deposits offer little evidence that declining prey resources were a primary cause of extinction for these large cats.

## Introduction

The saber-toothed cat, *Smilodon fatalis* and American lion, *Panthera atrox* were among the largest of the Pleistocene terrestrial carnivores; these, along with other megfauna were extinct by ∼12,000 years ago [1]. The cause of the terminal Pleistocene extinctions is debated, with potential extinction hypotheses including climate change, human overkill, and the synergistic combination of human effects (both direct and indirect) during a time of interglacial warming in North America (e.g., [1]–[4]). While large carnivores like *S. fatalis* and *P. atrox* are unlikely to have been directly hunted to extinction by humans, they were likely vulnerable given competition with humans for prey species [4]. It has been noted that when prey resources are limited, carnivores often consume carcasses more thoroughly and engage in durophagy (i.e., the processing of bone) [5]–[6]. Thus, we hypothesize that if prey became scarcer (either due to humans entering the carnivore guild or climate change, or both), competition might have forced large-bodied cats to consume both soft/tough tissues and less-preferred, harder bone.

The Rancho La Brea tar seep deposits in California, representing the past 50,000 years [7], are dominated by carnivorans and provide an abundance of remarkably well-preserved specimens of *S. fatalis* and *P. atrox* [8]–[9]. Previous work has suggested that times were tougher, or perhaps harder, at La Brea than for modern analogous carnivore guilds, as carnivores are inferred to have more fully utilized carcasses, based on incidences of tooth breakage [10]–[12]. Specifically, *P. atrox* has the greatest incidence of tooth breakage of canines (36%) while *S. fatalis* has the lowest incidence for extinct Pleistocene taxa (11.2%) [11]. These data contrast with those for extant large carnivorans, which have average canine breakage of ∼7%, including felid values averaging between 3.2% to 9.8% (in the jaguar, *Panthera onca* and leopard, *Panthera pardus*, respectively) and hyaenid values averaging between 8.3 to 9.6% (in the striped hyena, *Hyaena hyaena* and spotted hyena, *Crocuta crocuta*, respectively) [11]. While tooth breakage is used as a proxy for carcass utilization, it is also possible that increased tooth breakage in extinct taxa is a result of carnivorans taking down larger prey. While Van Valkenburgh and Hertel [10] exclude this explanation because both prey and predator sizes are larger during the Pleistocene, it is important to note that larger predators have relatively weaker teeth for a given tooth shape (e.g., canines can support a smaller percentage of an animal's body weight with increased body size) [13]–[15].

The analysis of the microscopic wear features left during the processing of food can also be used to infer extant and extinct diets including relative durophagy (e.g., [16]–[18]). High-resolution SEM-based microwear feature analysis demonstrates a lower frequency of pits and higher frequency of long scratches in carnivores that avoid bone [16]. And, *S. fatalis* from La Brea has lower incidence of pits and more scratches on carnassial teeth than the soft/tough-tissue specialist cheetah, *Acinonyx jubatus*, which suggests that the saber-toothed cat avoided bone and did not utilize carcasses completely [16]. In contrast, the canines of *S. fatalis* evince heavy pitting but few scratches compared with extant taxa ranging from cheetahs to spotted hyenas [17]. Conflicting interpretations of the inferred feeding behavior of *S. fatalis* from La Brea, based on microwear feature data and tooth breakage data, suggest that other methods are necessary to clarify degree of carcass utilization by carnivores at La Brea during the Pleistocene.

Dental Microwear Texture Analysis uses while-light confocal profilometry and scale-sensitive fractal analysis to quantify dental microwear, characterizing overall surface textures by anisotropy (*epLsar*), complexity (*Asfc*), scale of maximum complexity (*Smc*), textural fill volume (*Tfv*), and heterogeneity (*HAsfc*). Specifically, DMTA can help distinguish between hard- and soft- or tough-object consumption and has been used to distinguish dietary niches in primates, bovids, kangaroos, and carnivorans [18]–[24]. In addition, DMTA is an automated procedure that characterizes whole surfaces in 3D, thus reducing interobserver measurement error [19], [21], [25]–[28]. In carnivorans, taxa observed to avoid complete carcass utilization including shunning bone, e.g., cheetahs, can be distinguished from species with more generalized behavior (e.g., lions) and those that more fully utilize carcasses (e.g., hyenas) in having greater anisotropy, lower complexity, a higher scale of maximum complexity, and lower textural fill volume [18]. As these DMTA characters correspond to expectations based on the consumption of softer verses harder foods [29] by a diversity of mammals as mentioned above, we here examine dental microwear textures of extinct carnivorans from La Brea to clarify their feeding ecology and shed light on their extinction.

Specifically, we test the following hypotheses: (i) DMTA characters indicative of durophagy (i.e., greater *Asfc* and *Tfv*, lower *epLsar* and *Smc*) will be more extreme in extinct carnivorans during the Pleistocene, consistent with increased tooth breakage data, (ii) *S. fatalis* utilized carcasses less than *P. atrox*, potentially due to elongated canines in *S. fatalis* and consistent with tooth breakage data, and (iii) *S. fatalis* and *P. atrox* exhibit changes in DMTA characters through time (∼30–35 Ka to 11.5 Ka, mean calibrated ages; Ref. [7]) consistent with increased carcass utilization, due to declining prey availability.

## Results

### Species Comparisons

**Figure 1:**
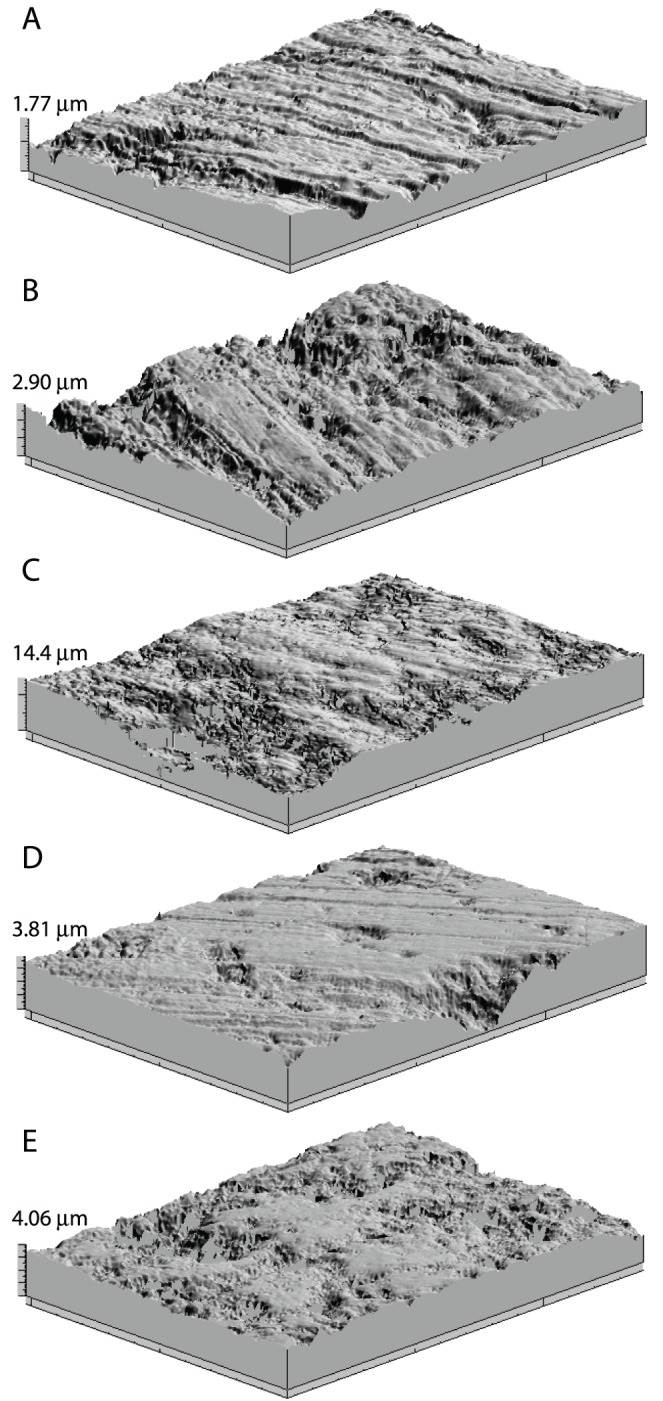
Three-dimensional photosimulations of microwear surfaces of all extant and extinct carnivorans analyzed. Examples include: (A) *A. jubatus* (AMNH 161139), (B) *P. leo* (USNM236919), (C) *C. crocuta* (AMNH 83592), (D) *P. atrox* (LACMHC 6996), and (E) *S. fatalis* (LACMHC 2002-298).

**Figure 2:**
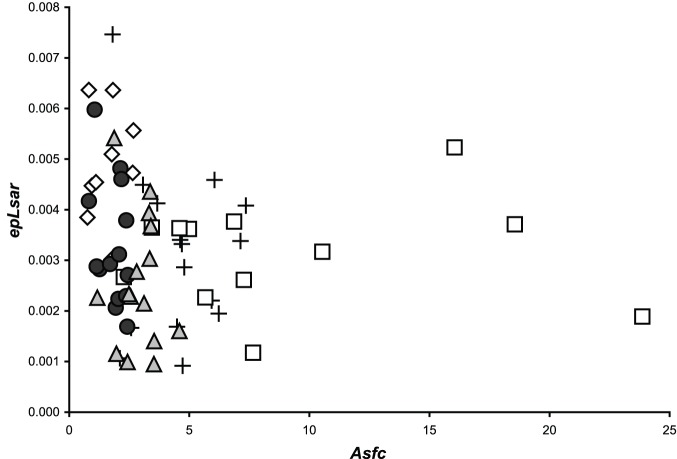
Bivariate plot of anisotropy and complexity of extant and extinct carnivorans. Extant cheetahs (*A. jubatus*, ◊), African lions (*P. leo*, +), and hyenas (*C. crocuta*, □) are from Ref. 18 and supplemental unpublished samples from wild caught animals throughout Africa. The extinct American lion (*P. atrox*, •) and saber toothed cat (*S. fatalis*, ▴) are from the Pleistocene Rancho La Brea site located in southern California (see Table S1).

Results are presented in Tables 1 and 2 and illustrated in Figures 1 and 2. Comparisons between *A. jubatus*, *P. leo*, and *C. crocuta* yield results consistent with those of Schubert et al. [18], as the majority of specimens analyzed are from the original paper; however, the addition of new specimens, minor corrections to prior typographic errors (noted in Table S1), and the addition of *HAsfc*_(9×9)_, does affect the means and other descriptive statistics of these taxa (Table 1).

Most notably, mean anisotropy (*epLsar*) is significantly greater in *A. jubatus* than in all extant and extinct taxa (Tables 1 and 2). In contrast, mean complexity (*Asfc*) averages are greatest in *C. crocuta*, followed by *P. leo*, *S. fatalis*, *P. atrox*, and *A. jubatus* (from greatest to least, Table 1). *Acinonyx jubatus* specimens have significantly lower complexity (*Asfc*) than either *P. leo* or *C. crocuta*. Additionally, Fisher’s LSD tests on complexity suggest *C. crocuta* differs from *P. leo* (Table S2). Both Fisher’s LSD and Tukey’s HSD tests on complexity further distinguish *S. fatalis* from all extant and extinct taxa (Table S2); however, *S. fatalis* is not significantly different from *P. leo* when using Dunn’s procedure (Table 2). *Panthera atrox*, on the other hand, is significantly different from all extant and extinct taxa except *A. jubatus* (Table 2). The scale of maximum complexity (*Smc*) is greatest in *P. leo* and *S. fatalis*; however, *Smc* distributions are highly skewed (Table 1) with mean values four to six times median values. In contrast, median *Smc* values are nearly identical in *P. leo* and *C. crocuta*, followed by *A. jubatus*, *S. fatalis*, and *P. atrox* (Table 1). All mean heterogeneity (*HAsfc*) values increase as the number of cells increases (i.e., 9 to 81), in all taxa. *HAsfc*_(3×3)_ and *HAsfc*_(9×9)_ mean values vary significantly between *A. jubatus* and *S. fatalis*, both of which consistently demonstrate the highest and lowest heterogeneity values (at both grid scales), respectively (Tables 1 and 2). *HAsfc*_(9×9)_ mean values can further distinguish *A. jubatus* from *P. atrox*, and *P. leo* from *S. fatalis* (Table 2) in addition to *P. leo* from *P. atrox* (when comparing ranked data; Table S2). Similar to mean *Asfc* values, mean texture fill volume (*Tfv*) is lowest in *A. jubatus*, followed by *P. atrox*, *S. fatalis*, *P. leo*, and *C. crocuta*, in ascending order (Table 1). Furthermore, *A. jubatus* has significantly lower mean *Tfv* than all extinct and extant taxa except *P. atrox.* And, *P. atrox* has significantly lower mean *Tfv* than all extinct and extant taxa except *A. jubatus.* Finally, *S. fatalis* mean *Tfv* is significantly greater than both *A. jubatus* and *P. atrox,* in contrast to the remainder of extant taxa (i.e., *P. leo* and *C. crocuta*).

**Table 1:** Descriptive statistics for each DMTA variable by species.

Taxon	Statistic	*n*	*Asfc*	*epLsar*	*Smc*	*Tfv*	*HAsfc_(3_*_×*3)*_	*HAsfc_(9_*_×*9)*_
*Acinonyx jubatus*	Mean	9	1.590	0.0049	0.286	5071	0.589	1.348
(extant)	Median		1.767	0.0047	0.209	2581	0.512	1.032
	Standard Deviation		0.737	0.0011	0.154	5372	0.278	0.895
	Skewness (Fisher)		0.424	−0.125	1.12	0.749	0.987	1.464
	*p* for normality (Shapiro-Wilk)		0.165	0.764	0.085	0.147	0.069	0.039
*Panthera leo*	Mean	15	4.616	0.0031	1.013	10413	0.471	0.895
(extant)	Median		4.690	0.0033	0.150	11358	0.442	0.799
	Standard Deviation		1.729	0.0017	2.596	4074	0.156	0.314
	Skewness (Fisher)		−0.080	0.981	3.566	−0.664	0.67	1.449
	*p* for normality (Shapiro-Wilk)		0.611	0.211	<0.0001	0.042	0.443	0.032
*Crocuta crocuta*	Mean	12	9.315	0.0031	0.151	12320	0.462	0.836
(extant)	Median		7.070	0.0034	0.151	14142	0.415	0.700
	Standard Deviation		6.708	0.0011	0.001	5666	0.18	0.333
	Skewness (Fisher)		1.215	0.035	0.504	−0.823	0.725	0.802
	*p* for normality (Shapiro-Wilk)		0.046	0.666	0.151	0.326	0.273	0.072
*Panthera atrox*	Mean	15	1.812	0.0033	0.562	6051	0.485	0.692
(extinct)	Median		2.049	0.0029	0.342	7063	0.451	0.643
	Standard Deviation		0.562	0.0012	0.661	4637	0.134	0.184
	Skewness (Fisher)		−0.527	0.949	3.201	0.096	0.721	0.869
	*p* for normality (Shapiro-Wilk)		0.046	0.183	<0.0001	0.069	0.202	0.111
*Smilodon fatalis*	Mean	15	2.900	0.0026	1.123	10213	0.396	0.666
(extinct)	Median		3.113	0.0023	0.267	12819	0.369	0.589
	Standard Deviation		0.845	0.0013	2.627	5460	0.108	0.225
	Skewness (Fisher)		−0.211	0.722	3.629	−0.701	0.654	1.509
	*p* for normality (Shapiro-Wilk)		0.671	0.337	<0.0001	0.105	0.352	0.007

*n,* number of individuals sampled*; Asfc*, area-scale fractal complexity; *epLsar*, anisotropy; *Smc*, scale of maximum complexity; *Tfv*, texture fill volume; *HAsfc*_(3×3)_, *HAsfc*_(9×9)_ heterogeneity of complexity in a 3×3 and 9×9 grid, respectively.

**Table 2:** Pairwise comparisons using Dunn’s procedure of extant and extinct taxa.

	C. crocuta	P. leo	P. atrox	S. fatalis
**Asfc**				
A. jubatus	−**41.6** *	**−32.3** *	−3.5	−**19.7** *
C. crocuta		9.3	**38.1** *	**21.9** *
P. leo			**28.7** *	12.6
P. atrox				−**16.1** *
**epLsar**				
A. jubatus	**22.4** *	**23.6** *	**20.6** *	**30.6** *
C. crocuta		1.2	−1.8	8.2
P. leo			−3.0	7.0
P. atrox				10.0
**Smc**				
A. jubatus	**21.9** *	13.2	−10.5	0.0
C. crocuta		−8.8	−**32.4** *	−**21.9** *
P. leo			−**23.7** *	−13.1
P. atrox				10.5
**Tfv**				
A. jubatus	−**26.3** *	−**18.4** *	−3.1	−**18.3** *
C. crocuta		7.9	**23.2** *	8.1
P. leo			**15.3** *	0.1
P. atrox				−**15.1** *
**HAsfc_(3_**_×**3)**_				
A. jubatus	9.7	6.8	4.6	**16.2** *
C. crocuta		−2.9	−5.1	6.5
P. leo			−2.2	9.4
P. atrox				11.6
**HAsfc_(9_**_×**9)**_				
A. jubatus	12.4	6.6	**19.8** *	**24.1** *
C. crocuta		−5.8	7.4	11.7
P. leo			13.3	**17.5** *
P. atrox				4.3

* Significant values (*p*<0.05) represent analyses performed absent of the Bonferroni correction (see methods). *Asfc*, area-scale fractal complexity; *epLsar*, anisotropy; *Smc*, scale of maximum complexity; *Tfv*, texture fill volume; *HAsfc*_(3×3)_, *HAsfc*_(9×9)_ heterogeneity of complexity in a 3×3 and 9×9 grid, respectively.

### Temporal Comparisons

Results are presented in Tables 3 and 4. Specifically, we compared like taxa across time by examining specimens from pits representing different ages (dates are noted in Table 3). Five specimens of *S. fatalis* were sampled per pit, in order from oldest to youngest (mean calibrated age, Ref. [7]): pit 91 (29,068, oldest), pit 3 (18,593, intermediate), and pit 67 (61–67 was dated, 11,581, youngest; Table 3). Five *P. atrox* specimens were sampled from each of the following temporal categories (mean calibrated age, Ref. [7]): pit 77 (35,370) and 91 (oldest), pit 3 and 4 (14,546, intermediate), and pit 67 (youngest). Although samples sizes were relatively low in each temporal category (*n* = 5), some differences are apparent (Tables 3 and 4).

**Table 3:** Mean, standard deviation (SD), and *p*-values for dental microwear characters (*Asfc, epLsar, Smc, Tfv, HAsfc_(3_*_×*3)*_ *, HAsfc_(9_*_×*9)*_) of extinct taxa from different temporal pits at Rancho La Brea, California.

		Pit 77,91		Pit 3,4		Pit 67		
Taxon	DMTA Character	mean	SD	mean	SD	mean	SD	*p*-value
*P. atrox*	*Asfc*	2.33	0.16	1.521	0.602	1.584	0.461	**0.025** *
	*epLsar*	0.0025	0.0008	0.0039	0.001	0.0034	0.0015	0.125
	*Smc*	0.271	0.047	1.104	0.985	0.312	0.067	**0.010** *
	*Tfv*	7338	5402	5852	3732	4962	5341	0.827
	*HAsfc_(3_*_×*3)*_	0.440	0.034	0.565	0.193	0.450	0.111	0.619
	*HAsfc_(9_*_×*9)*_	0.584	0.081	0.789	0.212	0.703	0.200	0.264
*S. fatalis*	*Asfc*	3.001	0.498	2.74	0.571	2.959	1.369	0.651
	*epLsar*	0.0031	0.0014	0.0031	0.0013	0.0015	0.0005	**0.050** *
	*Smc*	0.646	0.768	0.493	0.495	2.229	4.584	0.403
	*Tfv*	9533	5377	10446	6088	10658	6120	0.914
	*HAsfc_(3_*_×*3)*_	0.344	0.034	0.408	0.147	0.435	0.112	0.566
	*HAsfc_(9_*_×*9)*_	0.542	0.076	0.607	0.104	0.850	0.307	0.179

* Significant values (*p*<0.05; Kruskal-Wallis test). Mean calibrated ages in years before present (and standard deviations) for the pits studied are noted in order from oldest to youngest: Pit 77, 35370 (no SD reported); Pit 91, 29068 (18367); Pit 3, 18593 (5541); Pit 4, 14546 (7768); and, Pit 67 (61–67 was examined), 11581 (3768; all dates are taken from Ref. 7). *S. fatalis* consists of only specimens from Pits 3, 67, and 91; however, *P. atrox* required supplemental specimens from similarly aged pits to increase sample sizes, temporally. *Asfc*, area-scale fractal complexity; *epLsar*, anisotropy; *Smc*, scale of maximum complexity; *Tfv*, texture fill volume; *HAsfc*_(3×3)_, *HAsfc*_(9×9)_ heterogeneity of complexity in a 3×3 and 9×9 grid, respectively.

**Table 4:** Pairwise comparisons using Dunn’s procedure of extinct taxa from different pits spanning ∼35,000 years at Rancho La Brea, California.

	S. fatalis			P. atrox	
	Pit 3	Pit 91		Pit 3&4	Pit 91&77
**Asfc**					
Pit 67	2.6	1.0	Pit 67	−0.8	−**7.0** *
Pit 3		−1.6	Pit 3&4		−**6.2** *
**epLsar**					
Pit 67	**−6.0** *	**−6.0** *	Pit 67	**−**1.6	4.0
Pit 3		0.0	Pit 3&4		**5.6** *
**Smc**					
Pit 67	**−**2.2	**−**3.8	Pit 67	**−6.2** *	2.0
Pit 3		**−**1.6	Pit 3&4		**8.2** *
**Tfv**					
Pit 67	0.6	1.2	Pit 67	**−**0.2	**−**1.6
Pit 3		0.6	Pit 3&4		**−**1.4
**Hasfc_(3×3)_**					
Pit 67	1.2	3.0	Pit 67	**−**2.4	0.0
Pit 3		1.8	Pit 3&4		2.4
**Hasfc_(9×9)_**					
Pit 67	2.0	5.2	Pit 67	**−**2.0	2.6
Pit 3		3.2	Pit 3&4		4.6

* Significant values (*p*<0.05, critical value is 5.5) represent analyses performed absent of the Bonferroni correction (see methods).

*Asfc*, area-scale fractal complexity; *epLsar*, anisotropy; *Smc*, scale of maximum complexity; *Tfv*, texture fill volume; *HAsfc*_(3×3)_, *HAsfc*_(9×9)_ heterogeneity of complexity in a 3×3 and 9×9 grid, respectively.

*P. atrox* complexity is significantly lower in specimens from intermediate (3 and 4) and younger (67) pits, as compared to older (77 and 91) pits (Tables 3 and 4). In contrast, anisotropy is lowest in specimens from the oldest pits (77 and 91) and significantly lower than mean *epLsar* values from the intermediate pits (3 and 4). Scale of maximum complexity is significantly higher for intermediate pit samples (3 and 4) compared to older and younger pits (Tables 3 and 4). Overall, *S. fatalis* has similar DMTA mean values across time with one notable exception. Specifically, *S. fatalis* has significantly lower *epLsar* values in specimens from the youngest pit (67), in contrast to both pit 3 and pit 91(Tables 3 and 4).

## Discussion

### Species Comparisons

Dental microwear attributes most indicative of durophagy in extant taxa (i.e., greater *Asfc* and *Tfv*) are not especially pronounced in the analyzed extinct taxa. If *P. atrox* and *S. fatalis* more fully utilized carcasses we would expect them to have greater *Asfc* and *Tfv* than *P. leo* and *Asfc* and *Tfv* values in-line with *C. crocuta*; however, both mean *Asfc* and *Tfv* are lower than all extant taxa except *A. jubatus*. While *P. atrox* is distinct in *Asfc* and *Tfv* from extant taxa known to more fully utilize carcasses, *S. fatalis* is most similar in *Tfv* and *Asfc* to *P. leo*. Additionally, *P. atrox* has significantly lower *Asfc* and *Tfv* than *S. fatalis*, suggesting that *P. atrox* avoided bone more than *S. fatalis.* These DMTA results are inconsistent with interpretations of extremely high rates of tooth breakage in *P. atrox* (36% in canines), and lower, but still high rates in *S. fatalis* (11.2%) [11]. Thus, DMTA and tooth breakage data lead to seemingly conflicting interpretations of relative amounts of hard- and soft-tissue utilization. As extant taxa consistently maintain expected texture attributes (i.e., greater *Asfc* and *Tfv*, lower *epLsar* and *Smc*) and these characters are similarly used to infer the consumption of soft verses hard objects in bovids, primates, and marsupials (e.g., [18], [21]–[24]), we believe that dental microwear textures provide an appropriate proxy for extent of carcass utilization.

Tooth breakage data may reflect this too, but damage from food processing must be considered in combination with that from prey capture. While teeth can break from crushing bone, the stresses associated with chewing may be lower than those associated with prey capture (as bite forces are greater at canines as compared to carnassials) [30]–[31]. Further, when carnivores take down prey we expect canines to break more frequently than pre-carnassial premolars or carnassials. For example, in taxa that routinely scavenge and utilize carcasses heavily (e.g., Hyaenidae), canines and pre-carnassial premolars exhibit similar levels of tooth breakage (i.e., 8.93 and 8.73%, respectively) [11]. In contrast, when *A. jubatus* has broken teeth, they are at least twice as likely to occur in canines as carnassials [11], [32]. All extinct taxa including the dire wolf, *Canis dirus,* coyote, *Canis latrans,* gray wolf, *Canis lupus, S. fatalis,* and *P. atrox* have antemortem pre-carnassial premolar breakage of ∼3% (ranging from 3–3.5%) as compared to canine breakage of ∼21.5% (ranging from 11.2 to 36%) [11]. Thus, increased canine breakage in extinct carnivores may be consistent with damage incurred from greater forces exerted when capturing larger prey. While this idea has been thought unlikely due to the fact that both *S. fatalis* and *P. atrox* were also larger in size than analogous extant carnivorans [10], larger predators have relatively weaker teeth that can support declining prey sizes relative to a predator’s body size [14]. Because larger teeth are more vulnerable to fracture, tooth breakage is likely to increase in larger carnivores when trying to capture larger prey, all else being equal [13]–[15]. Based on measured and modeled data, canines from a fox-sized predator can support prey ∼7.3 times its body weight, in contrast to ∼4.4 times for a lion-sized predator, and 2.2 times for *Smilodon* with elongated canines [14]. Consistent with the idea that taking down prey can result in increased tooth breakage, female lions, which do a greater share of the hunting [33], have significantly greater tooth breakage than males (p<0.01) [32]. Similarly, female hyenas (*C. crocuta*) have significantly greater tooth breakage (at the 0.10 level) [32] than males and are also demonstrated to be larger in size and more aggressive than males [34]–[35]. Although greater tooth breakage occurs in smaller female lions and larger female hyenas, these differences likely have less to do with body size differences between males and females and instead result from behavioral differences including greater incidence of hunting or defense of carcasses. Increased canine tooth breakage in *P. atrox* and *S. fatalis* may therefore be indicative of the consumption of larger prey during the Pleistocene, with *P. atrox* potentially taking down larger prey or engaging in a higher frequency of prey captures (as compared to consuming prey that have already been killed) than *S. fatalis.*

*Smilodon fatalis* may have been a more catholic carnivore, both taking down prey and consuming carcasses already acquired by other individuals. Models of *S. fatalis* bite forces suggest they were relatively weak, approximately one-third those of *P. leo* [36], and lower than predicted by its body size [37]. Despite differences in bite force however, DMTA data are similar between these taxa and suggest that dietary behavior may have been similar, while other evidence suggests that prey capture techniques were likely different. For example, *S. fatalis* with a low bite force quotient is consistent with a ‘canine-shear bite’ that may have allowed for more efficient kills, as they are unlikely to have been able to immobilize prey with their teeth [36]–[38]. However, cortical thickening of the humeri in *S. fatalis* may have allowed them to better subdue prey using their forelimbs; subsequently reducing potential stresses and tooth breakage as compared to sympatric La Brea carnivores [39]. Additionally, *S. fatalis* is thought to have been social based on percent abundance data from La Brea [40]–[41] and may have displayed prey partitioning behaviors similar to *P. leo*, potentially introducing similar dynamics of carcass sharing. Thus, while morphological evidence suggests that prey were hunted in different ways, similar microwear suggests that degree of carcass utilization may have been similar.

Although DMTA data do not suggest that *S. fatalis* avoided bone more than the extant cheetah *A. jubatus* as opined by Van Valkenburgh and others [16], these differences may be due to methodological differences between studies. For example, while three-dimensional photosimulations of *C. crocuta* and *S. fatalis* appear similar in 2D, their depth scales are often distinct (ranging from 14.4 and 4.06 µm in Fig. 1C and 1D, respectively). It is difficult to characterize three-dimensional features in two-dimensions, and doing so may obfuscate differences in microwear indicative of different degrees of carcass utilization in extant carnivores [18].

Although less work has been done to understand the dietary behavior of *P. atrox*, morphological studies suggest that *P. atrox* had similar to slightly greater levels of sexual dimorphism than *P. leo* [42]. As *P. atrox* and its two closet relatives (the extinct European cave lion *Panthera spelaea* and *P. leo*) all display high levels of sexual dimorphism and likely traveled in groups, Meachen-Samuels and Binder [42] suggest that *P. atrox* did as well. However, if percent abundance is used as a proxy for social behavior, *P. atrox* was likely less social than the most abundant carnivores at La Brea, with only 2.6% abundance (similar to other large solitary animals including the short-faced bear and puma with ≤1% abundance), as compared to 33.3% in *S. fatalis* and 51.2% in *Canis dirus*, which are both inferred to be social [40]. Despite *P. atrox*’s large size (estimates range from 235 to 523 kg in males and 175 to 365 kg in females, the second largest carnivoran at La Brea second only to the short-faced bear *Arctodus simus*) [9], [43] and status as a conspecific of *P. leo*, its carcass utilization was evidently more like that of the cheetah. While speed and agility provide the cheetah with added advantages for prey capture, *P. atrox*’s inferred stealth and large size may have allowed it both to take down large prey and defend its kills against other carnivores, distinguishing it from *S. fatalis* in terms of avoidance of bone (as inferred from significantly lower *Tfv* and *Asfc* values). Additionally, if *P. atrox* was more solitary than its relatives, we would expect *P. atrox* to utilize carcasses to a lesser extent than social animals. As *P. atrox Asfc* and *Tfv* values are indistinguishable from those of *A. jubatus*, dietary behavior of *P. atrox* is likely more similar to cheetahs than its lion relatives. This interpretation is consistent with craniomandibular morphology that suggests that *P. atrox* was “no lion” [44]. However, anisotropy is lower in *P. atrox* than *A. jubatus,* suggesting that *P. atrox* may have been intermediate in dietary behavior between *A. jubatus* and *S. fatalis.*

Although La Brea is the most fossiliferous Pleistocene carnivore locality in North America, boasting large sample sizes of taxa less abundant elsewhere [8]–[9], inferred dietary behavior of extinct carnivorans there may not be entirely representative of these taxa throughout their distributions and over time. For example, the abundance of *S. fatalis* at La Brea may suggest heightened kleptoparasitism (i.e., the appropriation of a carcass from another predator) from the less abundant, although larger *P. atrox*, roughly analogous to occasional observations of lions appropriating carcasses from cheetahs today [45]. This sort of behavior could also explain why *P. atrox* and *S. fatalis* have microwear textures most similar to extant cheetahs and African lions, respectively; however, incidences of kleptoparasitism in cheetahs are typically infrequent (e.g., 3.5% in Kwandwe Private Game Reserve and 12% in Kruger National Park)[45]–[46]. Further, the lack of *P. atrox* individuals with *Asfc* values greater than 2.5 (approximately one-quarter to half the mean values of spotted hyenas and African lions, respectively; Table 1) suggests that durophagous behavior was probably rare in American lions even if kleptoparasitism occurred. While the extinct carnivores may have been provided with prey items from the tar pits for their last meal at La Brea (prior to succumbing themselves), dental microwear textures capture an animal’s past several meals and are likely representative of a carnivores diet well before death.

### Temporal Comparisons

Comparisons of the most abundant carnivorans, *C. dirus* and *S. fatalis,* through time suggest that body size changes may correlate with climatic fluctuations [47]–[50]. In contrast, other work by Prothero and authors [51] suggests stasis in both birds and mammals through time at La Brea. While temporal comparisons of body size data are equivocal, temporal comparisons of tooth breakage data from older (pit 3, ∼18,593+/−5,541 Ka) [7] to younger (pits 61/67, ∼11,581+/−3,768 Ka) [7] deposits yield minor but significant differences in incidence of tooth breakage over time in both *S. fatalis* and *C. dirus* [12]. Similarly, we observe minor differences in DMTA attributes over time with significantly lower anisotropy in *S. fatalis* occurring during the most recent deposit (pit 67) in contrast to the older deposits (pit 3 and pit 91, approximate age of pit 91 is 29,068+/−1 SD of 4,571; Tables 3 and 4) [7]. This might reflect a decline in consumption of excessively tough tissues, though a lack of higher complexity implies no greater consumption of bone (given caveats for small sample sizes). Temporal comparisons of *P. atrox* suggest that degree of carcass utilization declined over time, as complexity declined while anisotropy increased (Tables 3 and 4). Small samples sizes from each temporal pit and temporally mixed pits [7], [52]–[53] may both contribute to minimal differences in dental microwear textures. Nevertheless, temporal comparisons of *P. atrox* suggest lower carcass utilization during more recent times and are inconsistent with the idea that times got tougher during the late Pleistocene at La Brea.

Dental microwear textural data of the dire wolf are consistent with our felid data and also provide little support for the hypothesis that La Brea carnivores engaged in heavy carcass utilization during the Pleistocene [54]. Specifically, *C. dirus* from La Brea (spanning pits 91 to 61/67, ∼20 Ka) [7] shows little difference in dental textural properties to the modern gray wolf *Canis lupus* and is discordant with the durophageous African wild dog *Lycaon pictus* [54], despite having greater canine tooth breakage than all extant canids (including >4 times as many broken canines than *L. pictus*) [11]. While dire wolves were likely bone consuming canids based on dental microwear textures and morphology, they were not habitual bone crushers and likely engaged in such activities less than African hunting dogs [54]. Collectively, these data suggest that carcass utilization did not vary over time, and are not consistent with the idea that humans contributed to the extinction of large carnivorans via competition for prey items or declining prey resources at La Brea. If anything, carcass utilization may have actually declined, slightly, in *P. atrox*.

### Concluding Remarks

DMTA here suggests that extinct carnivorans at La Brea may have utilized carcasses less than do some carnivorans today. This idea is inconsistent with interpretations of high incidences of tooth breakage in extinct Pleistocene carnivorans from La Brea compared with extant taxa. We suggest that tooth breakage data may be recording damage from both carcass utilization and prey-capture, with greater tooth breakage occurring due to increased prey size. Lower mean values for DMTA attributes consistent with greater durophagy (i.e., *Asfc* and *Tfv*) in both *S. fatalis* and *P. atrox* compared with both *P. leo* and *C. crucuta*, suggest that the late Pleistocene at La Brea was not any “tougher” (or perhaps “harder”) than the African savanna is today. Further, dental microwear texture comparisons through time offer no evidence that carcasses were utilized consistently more over time, especially for *P. atrox.* Thus, DMTA provides no support for the idea that prey-resources became scarcer over time. While competition with humans for prey is unlikely to explain the extinction of *P. atrox* and *S. fatalis* via competition for prey resources at La Brea, further work is necessary to assess the situation at other sites. Collectively, there is no evidence for greater carcass utilization during the Pleistocene; however, high levels of anterior tooth breakage could instead result from hunting megafauna and/or conspecific competition at La Brea. Thus, times may have been "tough," but not as originally proposed.

## Materials and Methods

### Materials

Carnivorans included in this study consist of the following extant and extinct species: *Acinonyx jubatus* (*n* = 9), *Panthera leo* (*n* = 15), *Crocuta crocuta* (*n* = 12), *Panthera atrox* (*n* = 15), and *Smilodon fatalis* (*n* = 15). Most extant data are reported in Ref. [18]; however, additional specimens of *A. jubatus* (*n* = 2) and *P. leo* (*n* = 4) were analyzed, minor typographic errors corrected from the Table 2 of the original publication (see Table S1), and an additional heterogeneity of complexity measure was included in this study (*HAsfc_9×9_*; Table 1). All specimens examined in this study are housed in publicly accessible collections and were examined and molded while visiting respective museums. Extant specimens are housed at the American Museum of Natural History (AMNH), National Museum of Natural History (NMNH), and Iziko South African Museum (SAM). Extinct specimens are housed at the Los Angeles Museum of Natural History, Page Museum (LACMHC, Page Museum Hancock Collection; LACMRLP, Los Angeles County Museum - Pit 91), and were selected based on their stratigraphic contexts from pits ranging in mean calibrated ages of 35,370 to 11,581 years before present (all dates are taken from Ref. [7] and noted in Table 3). Five specimens per pit (3, 67, and 91) were sampled for *S. fatalis*; however, as five *P. atrox* specimens were not available from pits 3 and 91, supplemental specimens were added from similarly aged pits 4 and 77, respectively. Approximately one third of specimens for each species have broken canines and/or premolars; thus, our sample is representative of both individuals lacking and containing antemortem tooth breakage.

### Dental Microwear

The enamel region of the lower carnassial shearing facet of the m1 trigonid (following Refs. [16] and [18]) was examined on all specimens. The entire shearing facet was first cleaned with cotton swabs soaked in acetone to remove preservative (e.g., Butvar). Once the tooth was dry, a mold was made using polyvinylsiloxane dental impression material (President’s Jet regular body, Coltène-Whaledent Corp., Cuyahoga Falls, OH, USA). Tooth replicas were then prepared using Epotek 301 epoxy resin and hardener (Epoxy Technologies Corp., Billerica, MA, USA).

Dental microwear texture analysis (DMTA) was performed on all replicas that preserved antemortem microwear using while-light confocal profilometry and scale-sensitive fractal analysis (SSFA) [18]–[20]. All specimens were scanned in three dimensions in four adjacent fields of view, for a total sampled area of 204×276 µm^2^. All scans were analyzed using SSFA software (ToothFrax and SFrax, Surfract Corp., www.surfrait.com) to characterize tooth surfaces according to the following variables: (*i*) Complexity (*Asfc*), change in surface roughness with scale is used to distinguish taxa that consume hard, brittle foods from those that eat softer ones; (*ii*) Scale of maximum complexity (*Smc*), the fine-scale limit of the *Asfc* line, with greater *Smc* values for surfaces with fewer small features; (*iii*) Anisotropy (*epLsar*), the degree to which surfaces show a preferred orientation, such as the dominance of parallel striations (as might be formed by carnassial action in meat slicing given constraints to tooth-tooth movement during occlusion) having more anisotropic surfaces; (*iv*) Heterogeneity (*HAsfc*_(3×3),_ *HAsfc*_(9×9)_), the degree of variation in texture complexity across a surface, measured by quantifying variation in *Asfc* between subdivided samples (a 3×3 and 9×9 grid, totaling 9 to 81 subsamples, respectively); and, (*v*) Textural fill volume (*Tfv*), a measure of difference in volume filled by large (10 µm) and small (2 µm) diameter square cuboids (high values would indicate many deep features between these sizes) [18]–[21].

In the case of extant carnivorous taxa, increased complexity (*Asfc*) and decreased anisotropy (*epLsar*) are associated with increased durophagy [18]. Furthermore, greater textural fill volume (*Tfv*) occurs on surfaces with moderate sized, deep features (in contrast to fewer smaller features) and appears greater with increased durophagy [18].

### Statistical Analysis

As the majority of DMTA variables are not normally distributed (Shapiro-Wilk tests, Table 1) we used non-parametric statistical tests (Kruskal-Wallis) to compare differences among all taxa or pits for species and temporal comparisons, respectively. We used Dunn’s procedure [55] to conduct multiple comparisons (either between taxa or between like taxa across time) absent of the Bonferroni correction. As the Bonferroni correction is meant to reduce the likelihood of false positives (Type I errors) by taking into consideration the number of comparisons being made, it also increases the probability of false negatives (Type II errors) [56]–[57]. Furthermore, we do not want the number of extant and/or extinct comparisons to affect statistical differences between taxa; thus, the Bonferroni correction is not appropriate for our comparisons. Additionally, we compared ranked data for all species pairs using both Fisher (LSD) and Tukey (HSD) tests and report these results in Table S2, noting deviations from Dunn's procedure when appropriate in the text. These tests were run for consistency with previous microwear texture analyses, and to help balance risks of Type I and Type II errors. Significant results by Fisher’s (LSD) test may be considered suggestive, or of marginal significance.

## Supporting Information

Table S1 **All carnivoran specimens examined and dental microwear characters.**
(PDF)

Table S2 **Standardized differences between ranked data.**
(PDF)

## References

[1] Barnosky AD, Koch PL, Feranec RS, Wing SL, Shabel AB (2004) Assessing the causes of Late Pleistocene extinctions on the continents. Science 306: 70–75. 15459379 10.1126/science.1101476

[2] Martin PS, Klein RG (1984) *Quaternary Extinctions: A Prehistoric Revolution* (The University of Arizona Press).

[3] Wroe S, Field J, Fullagar R, Jermiin LS (2004) Megafaunal extinction in the late Quaternary and the global overkill hypothesis. Alcheringa 28: 291–331.

[4] Koch PL, Barnosky AD (2006) Late Quaternary Extinctions: State of the Debate. Annu Rev Eco Evol Syst 37: 215–250.

[5] Haynes G (1982) Utilization and Skeletal Disturbances of North American Prey Carcasses. Arctic 35: 266–281.

[6] Mondini M, Muñoz AS (2008) Pumas as taphonomic agents: a comparative analysis of actualistic studies in the Neotropics. Quat Int 180: 52–62.

[7] O’Keefe RF, Fet EV, Harris JM (2009) Compilation, calibration, and synthesis of faunal and floral radiocarbon dates, Rancho La Brea, California. Contrib Sci 518: 1–16.

[8] Merriam JC, Stock C (1932) *The Felidae of Rancho La Brea* (Carnegie Institution of Washington).

[9] Stock C, Harris JM (2001) *Rancho La Brea: A record of Pleistocene life in California* (Natural History Museum of Los Angeles).

[10] Van Valkenburgh B, Hertel F (1993) Tough times at La Brea: tooth breakage in large carnivores of the Late Pleistocene. Science 261: 456–459. 17770024 10.1126/science.261.5120.456

[11] Van Valkenburgh B (2009) Costs of carnivory: tooth fracture in Pleistocene and Recent carnivorans. Biol J Linn Soc Lond 96: 68–81.

[12] Binder WJ, Valkenburgh BV (2010) A comparison of tooth wear and breakage in Rancho La Brea sabertooth cats and dire wolves across Time. J Vertebr Paleontol 30: 255–261.

[13] Van Valkenburgh B, Ruff CB (1987) Canine tooth strength and killing behavior in large carnivores. J Zool 212: 379–397.

[14] Freeman PW, Lemen CA (2006) An experimental approach to modeling the strength of canine teeth. Papers in Natural Resources 12.

[15] Plavcan JM, Ruff CB (2008) Canine size, shape, and bending strength in primates and carnivores. Am J Phys Anthropol 136: 65–84. 18186502 10.1002/ajpa.20779

[16] Van Valkenburgh B, Teaford MF, Walker A (1990) Molar microwear and diet in large carnivores: inferences concerning diet in the sabretooth cat, *Smilodon fatalis* . J Zool 222: 319–340.

[17] Anyonge W (1996) Microwear on canines and killing behavior in large carnivores: saber function in *Smilodon fatalis* . J Mammal 77: 1059–1067.

[18] Schubert BW, Ungar PS, DeSantis LRG (2010) Carnassial micrower and dietary behavior in large carnivorans. J Zool 280: 257–263.

[19] Ungar PS, Brown CA, Bergstrom TS, Walkers A (2003) Quantification of dental microwear by tandem scanning confocal microscopy and scale-sensitive fractal analyses. Scanning 25: 185–193. 12926610 10.1002/sca.4950250405

[20] Scott RS, Ungar PS, Bergstrom TS, Brown CA, Grine FE, et al (2005) Dental microwear texture analysis shows within-species diet variability in fossil hominins. Nature 436: 693–695. 16079844 10.1038/nature03822

[21] Scott RS, Ungar PS, Bergstrom TS, Brown CA, Childs BE, et al (2006) Dental microwear texture analysis: technical considerations. J Hum Evol 51: 339–349. 16908052 10.1016/j.jhevol.2006.04.006

[22] Ungar PS, Merceron G, Scott RS (2007) Dental microwear texture analysis of Varswater Bovids and Early Pliocene paleoenvironments of Langebaanweg, Western Cape Province, South Africa. J Mamm Evol 14: 163–181.

[23] Prideaux GJ, Ayliffe LK, DeSantis LRG, Schubert BW, Murray PF, et al (2009) Extinction implications of a chenopod browse diet for a giant Pleistocene kangaroo. Proc Natl Acad Sci U S A 106(28): 11646–11650. 19556539 10.1073/pnas.0900956106 PMC2710660

[24] Scott J (2012) Dental microwear texture analysis of extant African Bovidae. Mammalia 76: 157–174.

[25] Grine FE, Ungar PS, Teaford MF (2002) Error rates in dental microwear quantification using scanning electron microscopy. Scanning 24: 144–153. 12074496 10.1002/sca.4950240307

[26] Galbany J, Martínez LM, López-Amor HM, Espurz V, Hiraldo O, et al (2005) Error rates in buccal-dental microwear quantification using scanning electron microscopy. Scanning 27: 23–29. 15712754 10.1002/sca.4950270105

[27] Ungar PS, Scott RS, Scott JR, Teaford MF (2008) Dental microwear texture analysis: historical perspectives and new approaches. eds Irish JD & Nelson GC (Cambridge University Press, Cambridge), Vol 53, 389–425.

[28] Milhacher MC, Beatty BL, Caldera-Siu A, Chan D, Lee R (2012) Error rates and observer bias in dental microwear analysis using light microscopy. Palaeontologica Electronica 15: 1–22.

[29] Van Valkenburgh B (1996) Feeding behavior in free-ranging, large African carnivores. J Mammal 77: 240–254.

[30] Therrien F (2005) Mandibular force profiles of extant carnivorans and implications for the feeding behaviour of extinct predators. J Zool 267: 249–270.

[31] Christiansen P, Adolfssen JS (2005) Bite forces, canine strength and skull allometry in carnivores (Mammalia, Carnivora). J Zool 266: 133–151.

[32] Van Valkenburgh B (1988) Incidence of tooth breakage among large, predatory mammals. Am Nat 131: 291–302.

[33] Schaller GB (1972) The Serengeti Lion: A Study of Predator Prey Relations. Chicago University Press, Chicago, IL.

[34] Frank LG (1986) Social organization of the spotted hyaena *Crocuta crocuta*. II. Dominance and reproduction. Anim Behav 34: 1510–1510.

[35] Watts HE, Tanner JB, Lundrigan BL, Holekamp KE (2009) Post-weaning maternal effects and the evolution of female dominance in the spotted hyena. Proc Biol Sci 276: 2291–2298. 19324728 10.1098/rspb.2009.0268 PMC2677617

[36] McHenry CR, Wroe S, Clausen PD, Moreno K, Cunningham E (2007) Supermodeled sabercat, predatory behavior in *Smilodon fatalis* revealed by high-resolution 3D computer simulation. Proc Natl Acad Sci USA 104: 16010–16015. 17911253 10.1073/pnas.0706086104 PMC2042153

[37] Wroe S, McHenry C, Thomason J (2005) Bite club: comparative bite force in big biting mammals and the prediction of predatory behaviour in fossil taxa. Proc Biol Sci 272: 619–625. 15817436 10.1098/rspb.2004.2986 PMC1564077

[38] Therrien F (2005) Feeding behaviour and bite force of sabretoothed predators. Zoo J Linn Soc 145: 393–426.

[39] Meachen-Samuels JA, Van Valkenburgh B (2010) Radiographs reveal exceptional forelimb strength in the sabertooth cat, *Smilodon fatalis* . PLoS ONE 5(7): e11412. 20625398 10.1371/journal.pone.0011412 PMC2896400

[40] Carbone C, Maddox T, Funston PJ, Mills MGL, Grether G, et al (2009) A comparison of tooth wear and breakage in Rancho La Brea sabertooth cats and dire wolves across time. Biol Lett 5: 81–85.18957359

[41] Van Valkenburgh B, Maddox T, Funston PJ, Mills MGL (2009) Sociality in Rancho La Brea *Smilodon*: arguments favor ‘evidence’ over ‘coincidence.’. Biol Lett 5: 563–564. 10.1098/rsbl.2008.0526 PMC2657756 18957359

[42] Meachen-Samuels JA, Binder WJ (2010) Sexual dimorphism and ontogenetic growth in the American lion and sabertoothed cat from Rancho La Brea. J Zool 280: 271–279.

[43] Anyonge W (1993) Body mass in large extant and extinct carnivores. J Zool 231: 339–339.

[44] Christiansen P, Harris JM (2009) Craniomandibular morphology and phylogenetic affinities of *Panthera atrox*: implications for the evolution and paleobiology of the lion lineage. J Vertebr Paleontol 29: 934–945.

[45] Bissett C, Bernard RTF (2006) Habitat selection and feeding ecology of the cheetah (*Acinonyx jubatus*) in thicket vegetation: is the cheetah a savanna specialist? J Zool 271: 310–317.

[46] Mills MGL, Broomhall LS, du Toit JT (2004) Cheetah *Acinonyx jubatus* feeding ecology in the Kruger National Park and a comparison across African savanna habitats: is the cheetah only a successful hunter on open grassland plains? Wildl Biol 10: 177–186.

[47] Nigra JO, Lance JF (1947) A statistical study of the metapodials of the dire wolf group. Bull South Acad Sci 46: 26–34.

[48] Shaw CA, Tejado-Flores AE (1985) Biomechanical implications of the variation in *Smilodon* ectocuneiforms from Rancho La Brea. Contrib Sci 359: 1–8.

[49] O’Keefe FR (2008) Population-level response to the dire wolf, *Canis dirus*, to climate change in the Upper Pleistocene. J Vertebr Paleontol 28(3, Supplement): 122A.

[50] Meachen JA, Samuels JX (2012) Evolution in coyotes (*Canis latrans*) in response to the megafaunal extinctions. Proc Natl Acad Sci USA 109: 4191–4196. 22371581 10.1073/pnas.1113788109 PMC3306717

[51] Prothero DR, Raymond KR, Syverson V, Molina S (2009) Stasis in late Pleistocene birds and mammals from La Brea tar pits over the last glacial-interglacial cycle. Cincinnati Museum Center Scientific Contributions 3: 291–292.

[52] Behrensmeyer AK, Hook RW (1992) Paleoenvironmental contexts and taphonomic modes. In: Behrensmeyer AK, Damuth JD, DiMichele WA, Potts R, Sues HD, Wing SL, editors. Terrestrial Ecosystems Through Time. Chicago: University of Chicago Press. 15–136.

[53] Friscia AR, Van Valkenburgh B, Spencer L, Harris J (2008) Chronology and Spatial Distribution of Large Mammal Bones in Pit 91, Rancho La Brea. Palaios 23: 35–42.

[54] Schmitt E (2011) Analysis of bone crushing behaviro of the dire wolf (*Canis dirus*) using dental microwear texture analysis. East Tennessee State University. ProQuest Dissertations and Theses: 875882725.

[55] Dunn OJ (1964) Multiple Comparisons Using Rank Sums. Am Soc Qual 6: 241–252.

[56] Cabin RJ, Mitchell RJ (2000) To Bonferroni or Not to Bonferroni: When and How Are the Questions. Bull Ecol Soc Am 81: 246–248.

[57] Nakagawa S (2004) A farewell to Bonferroni: the problems of low statistical power and publication bias. Behav Ecol 15(6): 1044–1045.

