# Supplementary material for: Implications of Diet for the Extinction of Saber-Toothed Cats and American Lions
Source: PLoS One. 2012 Dec 26;7(12):e52453. doi: 10.1371/journal.pone.0052453 (PMC3530457; doi:10.1371/journal.pone.0052453)
Supplement: Table S1 — All carnivoran specimens examined and dental microwear characters. (PDF) [file pone.0052453.s001.pdf]

Table S1. All carnivoran specimens examined and dental microwear characters.

Taxon	Status/Pit	Museum	ID	*Asfc*	*epLsar*	*Smc*	*Tfv*	*HAsfc*$_{(3x3)}$	*HAsfc*$_{(9x9)}$	Data
Acinonyx jubatus	extant	AMNH	27897	1.821	0.0064	0.151	8347	0.952	1.698	S
		AMNH	119654	0.942	0.0045	0.341	11050	0.512	1.032	S
		AMNH	119656	0.817	0.0064	0.418	1618	0.399	0.767	S
		AMNH	119657	1.111	0.0045	0.209	38	0.543	2.237	S
		AMNH	161139	0.759	0.0038	0.150	0	0.739	1.070	S
		SAM	36849	2.645	0.0047	0.152	534	1.090	3.257	S
		SAM	38624	2.674	0.0056	0.208	6833	0.373	0.734	S
		USNM	161922	1.767	0.0030	0.601	2581	0.345	0.799	U
		USNM	540001	1.772	0.0051	0.345	14638	0.344	0.539	U
Crocuta crocuta	extant	AMNH	20809	6.867	0.0038	0.150	16323	0.372	0.627	S
		AMNH	83591	2.280	0.0027	0.151	1234	0.604	1.314	S
		AMNH	83592	23.864	0.0019	0.152	15255	0.384	0.672	S
		AMNH	187771	16.047	0.0052	0.150	19699	0.250	0.581	C
		AMNH	187772	4.988	0.0036	0.151	10135	0.320	0.556	C
		AMNH	187774	7.273	0.0026	0.151	11482	0.532	1.014	C
		SAM	33341	5.669	0.0023	0.150	13029	0.790	1.435	C
		SAM	33432	4.594	0.0036	0.151	7761	0.507	0.829	C
		SAM	36871	10.538	0.0032	0.150	15994	0.262	0.458	C
		SAM	40361	18.553	0.0037	0.150	17531	0.446	0.575	C
		SAM	83593	7.658	0.0012	0.151	15610	0.328	0.729	C
		SAM	38817b	3.445	0.0036	0.151	3783	0.752	1.243	C
Panthera leo	extant	AMNH	17274	4.690	0.0033	0.150	7463	0.416	0.784	C
		AMNH	39870	4.792	0.0029	0.150	12095	0.638	1.068	S
		AMNH	52072	7.354	0.0041	0.150	8860	0.513	0.826	S
		AMNH	81830	4.723	0.0009	0.151	10966	0.633	1.706	S
		AMNH	81836	6.227	0.0019	0.151	13425	0.535	0.829	S
		SAM	3983	5.929	0.0022	0.153	11358	0.831	1.357	S
		SAM	14893	1.807	0.0075	2.418	14934	0.417	0.693	C
		SAM	36873	6.050	0.0046	0.150	14769	0.511	1.158	S
		SAM	36874	4.617	0.0034	0.150	3852	0.541	0.951	S
		SAM	38222	7.132	0.0034	0.151	14318	0.309	0.799	S
		SAM	39302	2.110	0.0011	0.816	10307	0.442	0.783	S
		USNM	182297	2.582	0.0017	0.150	3710	0.263	0.535	U
		USNM	216602	4.487	0.0017	0.150	3774	0.266	0.598	U

		USNM	236919	3.075	0.0045	0.150	11433	0.427	0.630	U
		USNM	236920	3.665	0.0041	10.147	14929	0.320	0.701	U
Panthera atrox	pit 67	LACMHC	582	1.701	0.0029	0.267	762	0.508	0.725	U
		LACMHC	586	1.059	0.0060	0.417	11244	0.365	0.509	U
		LACMHC	6985	1.938	0.0021	0.342	930	0.601	0.855	U
		LACMHC	6996	1.145	0.0029	0.267	1530	0.451	0.936	U
		LACMHC	17025	2.076	0.0031	0.266	10345	0.324	0.492	U
	pit 4	LACMHC	593	2.132	0.0048	1.083	10158	0.306	0.557	U
		LACMHC	6991	2.175	0.0046	0.708	7457	0.414	0.643	U
		LACMHC	6993	1.262	0.0028	0.417	7252	0.746	0.831	U
	pit 3	LACMHC	587	0.822	0.0042	0.508	475	0.664	1.108	U
		LACMHC	597	1.213	0.0029	2.806	3920	0.695	0.807	U
	pit 91	LACMRLP	R15404	2.417	0.0017	0.343	341	0.388	0.577	U
		LACMRLP	R52033	2.376	0.0038	0.209	12683	0.463	0.719	U
	pit 77	LACMHC	595	2.049	0.0022	0.267	12617	0.448	0.578	U
		LACMHC	17031	2.371	0.0023	0.267	7063	0.429	0.508	U
		LACMHC	53639	2.438	0.0027	0.266	3984	0.473	0.536	U
Smilodon fatalis	pit 67	LACMHC	2002-104	1.173	0.0023	0.267	3781	0.420	0.902	U
		LACMHC	2002-213	4.590	0.0016	0.150	17830	0.344	0.556	U
		LACMHC	2002-234	3.529	0.0009	0.150	14090	0.606	1.220	U
		LACMHC	2002-293	1.968	0.0012	10.428	12819	0.473	1.053	U
		LACMHC	2002-298	3.537	0.0014	0.150	4772	0.329	0.519	U
	pit 3	LACMHC	2002-60	2.560	0.0023	0.267	12956	0.463	0.714	U
		LACMHC	2002-272	1.870	0.0054	0.416	190	0.285	0.592	U
		LACMHC	2002-622	3.349	0.0030	0.208	9884	0.232	0.452	U
		LACMHC	2002-828	2.808	0.0028	1.366	13621	0.588	0.691	U
		LACMHC	29184	3.113	0.0021	0.208	15579	0.474	0.589	U
	pit 91	LACMRLP	R29082	2.428	0.0010	2.015	7642	0.287	0.542	U
		LACMRLP	R29093	3.398	0.0037	0.266	11232	0.343	0.530	U
		LACMRLP	R30102	2.484	0.0023	0.266	1066	0.371	0.548	U
		LACMRLP	R36452	3.368	0.0044	0.417	13831	0.351	0.438	U
		LACMRLP	R39815	3.325	0.0039	0.267	13894	0.369	0.652	U

Status/pit, if extinct only the pit is noted. All pits are from the La Brea Tar Pits, Los Angeles, California, USA. *Asfc*, area-scale fractal complexity; *epLsar*, anisotropy; *Smc*, scale of maximum complexity; *Tfv*, texture fill volume; $HAsfc_{(3x3)}$, $HAsfc_{(9x9)}$ heterogeneity of complexity in a 3x3 and 9x9 grid, respectively. Data, notes the source of respective data according to the following: S, published in Ref. 18; C, corrected from Ref. 18; U, unpublished data new to this study.
