# Supplementary material for: Implications of Diet for the Extinction of Saber-Toothed Cats and American Lions
Source: PLoS One. 2012 Dec 26;7(12):e52453. doi: 10.1371/journal.pone.0052453 (PMC3530457; doi:10.1371/journal.pone.0052453)
Supplement: Table S2 — Standardized differences between ranked data. (PDF) [file pone.0052453.s002.pdf]

Table S2. Standardized differences between ranked data.

	C. crocuta	*P. leo*	*P. atrox*	*S. fatalis*
Asfc				
A. jubatus	**8.0****	**6.5****	0.7	**4.0****
C. crocuta		**2.0***	**8.4****	**4.8****
P. leo			**6.7****	**2.9****
P. atrox				**3.8****
epLsar				
A. jubatus	**2.9****	**3.2****	**2.8****	**4.2****
C. crocuta		0.2	0.3	1.2
P. leo			0.4	1.1
P. atrox				1.5
Smc				
A. jubatus	**3.1****	2.0	1.6	0.0
C. crocuta		1.4	**5.3****	**3.1****
P. leo			**4.1****	**2.3***
P. atrox				1.8
Tfv				
A. jubatus	**3.5****	**2.5***	0.4	**2.5***
C. crocuta		1.2	**3.5****	1.2
P. leo			**2.4***	0.0
P. atrox				**2.4***
$HAsfc_{(9x9)}$				
A. jubatus	1.6	0.9	**2.6***	**3.2****
C. crocuta		0.8	1.1	1.7
P. leo			**2.0***	**2.7***
P. atrox				0.7

*Significant values based on Fisher's LSD test are noted in bold text ($p<0.05$; critical value is 2.0).

**Significant values ($p<0.05$) based on both Fisher's LSD and Tukey's HSD tests (critical value is 2.81). *Asfc*, area-scale fractal complexity; *epLsar*, anisotropy; *Smc*, scale of maximum complexity; *Tfv*, texture fill volume; $HAsfc_{(9x9)}$ heterogeneity of complexity in a 9x9 grid, $HAsfc_{(3x3)}$ values are excluded as there are no significant differences between any extant or extinct taxa.
